# Convolutional Neural Network Based on Extreme Learning Machine for Maritime Ships Recognition in Infrared Images

**DOI:** 10.3390/s18051490

**Published:** 2018-05-09

**Authors:** Atmane Khellal, Hongbin Ma, Qing Fei

**Affiliations:** 1 School of Automation, Beijing Institute of Technology, Beijing 100081, China; feiqing@bit.edu.cn; 2 State Key Laboratory of Intelligent Control and Decision of Complex Systems, Beijing Institute of Technology, Beijing 100081, China

**Keywords:** classification, convolutional neural network, ensemble, extreme learning machine, features extraction, infrared images, maritime ships recognition, VAIS dataset

## Abstract

The success of Deep Learning models, notably convolutional neural networks (CNNs), makes them the favorable solution for object recognition systems in both visible and infrared domains. However, the lack of training data in the case of maritime ships research leads to poor performance due to the problem of overfitting. In addition, the back-propagation algorithm used to train CNN is very slow and requires tuning many hyperparameters. To overcome these weaknesses, we introduce a new approach fully based on Extreme Learning Machine (ELM) to learn useful CNN features and perform a fast and accurate classification, which is suitable for infrared-based recognition systems. The proposed approach combines an ELM based learning algorithm to train CNN for discriminative features extraction and an ELM based ensemble for classification. The experimental results on VAIS dataset, which is the largest dataset of maritime ships, confirm that the proposed approach outperforms the state-of-the-art models in term of generalization performance and training speed. For instance, the proposed model is up to 950 times faster than the traditional back-propagation based training of convolutional neural networks, primarily for low-level features extraction.

## 1. Introduction

The object recognition module of Autonomous sea Surface Vessels (ASVs) plays an essential role in safe maritime navigation. The ASV is required to recognize other nearby vessels, usually by using cameras guided with a Radar [1]. This procedure may operate properly in the day when the lighting conditions are favorable, but this is not the case at night. Therefore, including an infrared sensor such as thermal cameras, which are sensitive to thermal radiations, will further enhance the recognition performance. Thus, our work aims to develop an efficient recognition system able to recognize maritime ships, during both the day and night, using infrared images.

The earlier research works on maritime ships recognition apply traditional computer vision techniques. In [2], for example, a segmentation algorithm based on the Hough transform is utilized to locate the waterline of the ship, and then a 32-feature vector is computed using some information concerning the size, location, and structure of the ship. Next, the classification is conducted using a simple classifier, such as the K-Nearest Neighbor (KNN), linear, and quadratic classifiers. In [3], a useful features vector based on different characteristics is extracted and then classified using the Support Vector Machine (SVM) classifier.

However, the current research trend is employing machine learning algorithms to learn useful features instead of hand-designing them. In fact, since the emergence of Convolutional Neural Networks (CNNs) [4] in the ImageNet [5] challenge, they have become the favorable solution for general object recognition problems such as classification, localization, and detection. For this reason, convolutional neural networks have been widely adopted in object recognition systems in the visible domain, which has extended to the infrared thermal based recognition systems. For instance,  convolutional neural networks have been applied to pedestrian detection in the infrared spectrum [6,7,8], where enough data are available to train a tiny CNN architecture. As for maritime ships recognition research, a pre-trained CNN on the ImageNet is applied for features extraction in the case of [1]. In addition, a Fully Convolutional Network (FCN) is used for sea-land segmentation in the context of remote sensing, where the model is trained on Synthetic Aperture Radar (SAR) images [9].

Convolutional neural networks are trained by back-propagating the classification error, which requires a significant amount of training data depending on the network size. However, in the case of maritime ships, the available amount of training examples is very small. For example, to the best of our knowledge, VAIS dataset [1] is the largest publicly available dataset suitable for ships recognition research and contains only few hundred training samples. Fine-tuning of a pre-trained model yields poor performance, as discussed in [1], because of the problem of overfitting due to the lack of training data, even when using regularization techniques. Therefore, to overcome these problems, we propose a new approach fully based on Extreme Learning Machine (ELM) [10,11] to learn useful CNN features and perform a fast and accurate classification, which is suitable for small dataset problems.

The proposed approach is based on two components; first, features extraction using Extreme Learning Machine based training of Convolutional Neural Network, named ELM-CNN; and, second, features classification using an ensemble of extreme learning machines. The ELM-CNN algorithm is an unsupervised procedure to train any convolutional architecture based on the concept of ELM auto-encoding [12,13], while the ELM-based ensemble is a training algorithm that leans automatically how to combine the different outputs of ELM classifiers to improve the classification performance. The parameters of both models are computed by minimizing not only the classification error (reconstruction error in the case of the ELM-CNN) but also the network weights which leads to better generalization performance according to Bartlett’s theory [14].

The experimental results on VAIS dataset confirm that the proposed approach outperforms the state-of-the-art models in term of generalization performance. Moreover, the separate evaluation of the proposed approach components shows that the ELM-CNN algorithm outperforms the traditional back-propagation training of convolutional neural networks (in this paper, we refer to it as BP-CNN) in terms of generalization performance and training speed. Especially for low-level features extraction, the ELM-CNN is up to 950 times faster than the BP-CNN. As for the second component of the proposed approach, the experiments reveal that the ELM based ensemble in some cases improves the generalization performance by more than 16% and seven times faster than some traditional classifiers such as SVM, KNN, and ensembles such as bagged and boosted Decision Trees. The characteristics of the proposed approach, generalization performance, and training speed make it suitable for any object recognition problem in general, and maritime ships recognition in particular. Mainly when the training dataset is small, where the traditional BP-CNN fails due to the problem of overfitting, the proposed model performs efficiently.

The main contributions of our research can be summarized in the following points. (1) To the best of our knowledge, in this paper, we present the first work that uses an accurate and fast approach fully based on extreme learning machine for maritime ships recognition in the infrared spectrum. (2) A fast unsupervised learning algorithm to train any convolutional neural network for features extraction is introduced. (3) An efficient ELM based ensemble for classification, which learns automatically how to combine different ELM classifiers to enhance the generalization performance, is proposed. (4) An extensive visual analysis of the misclassified samples and some interpretations that may highlight forward perspectives are discussed.

Finally, the remainder of this paper is organized as follows. In Section 2, we describe our proposed approach, starting with an overall description, and then each component. The ELM-CNN algorithm for features extraction and the ELM based ensemble for classification are introduced in detail. In Section 3, the proposed framework is experimentally evaluated by performing an overall evaluation as well as separate evaluations of each approach component. Then, extensive discussions of the different aspects of the proposed models are presented in Section 4. Lastly, the article is concluded in Section 5.

## 2. Proposed Approach

To tackle the problems of availability of data, training speed, and generalization performance improvement, an approach fully based on extreme learning machine is introduced in this section. First, the overall description is presented, and then the detailed description of each component is provided.

### 2.1. Pipeline of the Proposed Approach

**Figure 1:**
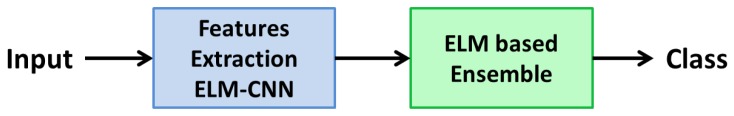
The proposed approach is built on two components: features extraction using the proposed Extreme Learning Machine based Convolutional Neural Network algorithm (ELM-CNN), and classification using the Extreme Learning Machine (ELM) based ensemble.

As shown in Figure 1, the proposed model is divided into two components: features extraction, and classification. For a given input image, first, convolutional neural network based features are extracted, and then it is classified into one of the classes.

#### 2.1.1. Features Extraction

To extract useful features, we have used a convolutional neural network structure. The CNN features are computed using a convolutional neural network trained with an unsupervised algorithm called ELM-CNN, based on extreme learning machine. The ELM-CNN is a unified framework to train any convolutional architecture, which is described in detail in Section 2.4.

#### 2.1.2. Classification

To improve the classification performance, we used an ensemble of extreme learning machine based classifiers. The proposed approach is a fully learnable ensemble that learns automatically how to combine different classifiers by applying the ELM algorithm. The model is described in Section 2.3.

In the following subsections, the proposed approach is described in detail; first, for a better understanding, we start with an ELM overview, and then the ELM based ensemble for classification is presented. Finally, the ELM based training algorithm for CNN is described.

### 2.2. Extreme Learning Machine Overview

Extreme Learning Machine (ELM) is a learning algorithm, initially introduced to train a Single Layer Feedforward Neural network [10,11]. In ELM theory, the input weights are randomly generated according to any continuous distribution function, while the output weights are analytically computed by the minimum norm solution of a linear system. A simple version of the ELM training procedure is described in Algorithm 1.

X is an N×D matrix that contains *N* training examples of dimension *D*; W is a D×L matrix that represents the interconnections between the input layer and the hidden layer; and b is an N×L matrix that represents a duplication of the bias vector (in our work, W and b are randomly generated according to the normal distribution). H is an N×L matrix called hidden matrix, while G(·) is a nonlinear piecewise continuous function that satisfies the ELM universal approximation capability theorems [15] (in this paper, the sigmoid function is used). β is an L×C matrix called output weights matrix, while $H^{†}$ stands for the Moore–Penrose generalized inverse of matrix H. T is an N×C matrix called the target matrix, which corresponds to target vectors (labels) in One-Hot encoding format of all training samples, while *C* represents the number of classes. ELM based auto-encoder (ELM-AE) [12] is an unsupervised algorithm used to learn the input weights by reconstructing the input. In other words, the target matrix is simply replaced with the input data matrix T=X.

**Algorithm 1** The ELM algorithm.**Input:** Dataset X, target T, and the number of hidden nodes *L*. **Output:** ELM parameters. Generate randomly the input weights and the bias W and b. Compute the hidden matrix $H=G\left(XW+b\right)$. Compute the output weights $\beta =H^{†}T$. Return the ELM parameters W, b, and β.

Note that β is the minimum norm solution ($\min_{\beta }\left(∥\beta ∥+∥H\beta -T∥\right)$), that minimizes both the classification error and the norm of weights. Thus, according to Bartlett’s theory, a network with small weights leads to better generalization performance [14]. In fact, the extreme learning machine is characterized by three intrinsic features: training speed, universal approximation capability, and good generalization performance. For these reasons, we chose the ELM as a base classifier in our ELM based ensemble, which is the subject of the next section.

### 2.3. ELM Based Ensemble for Classification

To improve the performance of a single model, and make full use of multiple models, we introduce an ensemble based on ELM. The described algorithm (Algorithm 2) consists of *M* independent ELM models, where the final decision is computed by combining the different ELM outputs with a set of parameters F learned by an ELM algorithm.

**Algorithm 2** Training of the ELM based ensemble for classification.**Input:** Dataset X, target T, number of individual models *M*, and number of hidden nodes *L*. **Output:** ELM based ensemble parameters. **for**
m=1
*M*
**do**  Generate randomly the input weights and biases $W^{\left(m\right)}$ and $b^{\left(m\right)}$.  Compute the hidden matrix $H^{\left(m\right)}=G\left(XW^{\left(m\right)}+b^{\left(m\right)}\right)$.  Compute the output weights $\beta^{\left(m\right)}={\left(H^{\left(m\right)})\right)}^{†}T$.  Compute the outputs $O^{\left(m\right)}=H^{\left(m\right)}\beta^{\left(m\right)}$. **end for** Compute the global hidden matrix $H_{g}=\left[O^{\left(1\right)}O^{\left(2\right)}\cdots O^{\left(M\right)}\right]$. Compute the fusion parameters $F=H_{g}^{†}T$. Return the ensemble parameters $W^{\left(m\right)}$, $b^{\left(m\right)}$, $\beta^{\left(m\right)}$, for m=1,2,⋯,M and F.

$O^{\left(m\right)}$ is the output of the $m^{th}$ model, and $H_{g}$ is the global hidden matrix. If all ELM models have the same size, then $H_{g}$ is an N×MC matrix. We compute the fusion parameters as the output weights of the global ELM model $F=H_{g}^{†}T$. It is an MC×C matrix and represents the combination weights of *M* different ELM models that contribute to the final decision. As for $H_{g}^{†}$ is the Moore–Penrose generalized inverse of matrix $H_{g}$. At this stage, the ELM based ensemble is entirely defined by its parameters $W^{\left(m\right)}$, $b^{\left(m\right)}$ and $\beta^{\left(m\right)}$, for m=1,2,⋯,M and F.

According to ELM theory, F is the solution that minimizes the classification error and the output weights norm ($\min_{F}\left(∥F∥+∥H_{g}F-T∥\right)$). Thus, under Bartlett’s theory, it will lead to better generalization performance [14].

**Figure 2:**
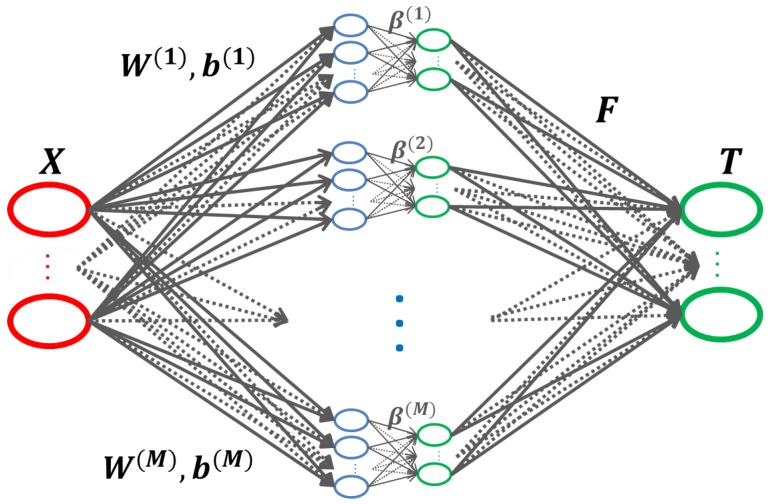
The proposed ELM based ensemble can be seen as a two hidden layers neural networks, trained using the ELM algorithm.

As shown in Figure 2, the ELM based ensemble can be viewed as a two hidden layers neural networks, where the first hidden layer has (M·L) nodes, and the second hidden layer contains (M·C) nodes. The advantages of the ELM based ensemble, such as the training speed, the network architecture, and generalization performance, make it very suitable for our application which is maritime ships recognition. Furthermore, providing better features as input to the ensemble will boost the performance. Therefore, in the next section, an algorithm based on ELM is described to train any convolutional structure which can be used for CNN features extraction.

### 2.4. ELM Based Training of Convolutional Neural Network

In this section, we explain the method of training a convolutional neural network using extreme learning machine algorithm (ELM-CNN). Any basic CNN model has three components: convolution (CONV), pooling (POOL), and fully connected layers (FC). In this paper, we treat the fully connected layer as a convolutional layer (FC layer can be viewed as 1×1 convolutional filters). Therefore, only CONV layers need to be trained, and for POOL layers, no parameters require to be tuned.

**Figure 3:**
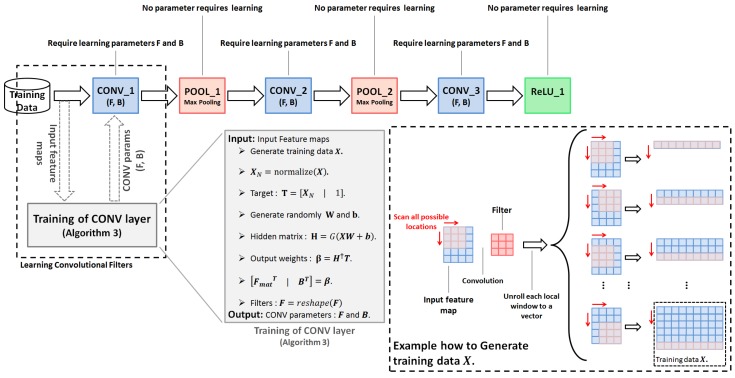
The overall structure of the ELM-CNN approach. Any basic CNN architecture can be implemented as a stack of blocks with a specified operation (Convolution, Pooling, and ReLU), where only the convolutional layers require training.

As shown in Figure 3, the convolutional neural networks can be implemented as a stack of blocks with a specified operation (convolution, pooling, and non-linearity). Each block has an input feature map, which is the output feature map of the previous layer (for the first layer, the input feature map is the input training dataset), and an output feature map (except for the last layer that produces the class scores).

In the training phase, for a given CNN architecture, we randomly initialize all the filters. Next, the data are propagated through the network by directly computing the output of each block. If the block is a CONV layer, first, the training is performed, and then the output can be evaluated after learning the convolutional kernels.

**Figure 4:**
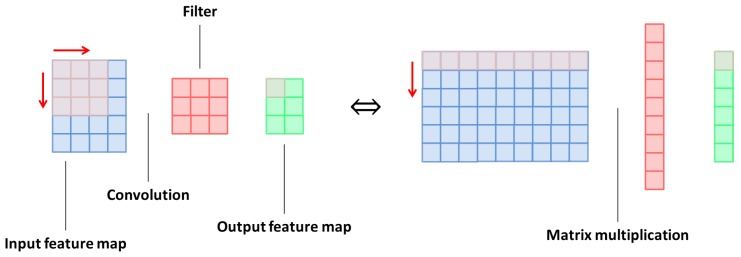
The convolution operation can be regarded as an element-wise multiplication of the convolutional filter with its corresponding local window in the input feature map.

The convolution operation can be seen as an element-wise multiplication of a filter (kernel) with its corresponding local window (receptive field) in the input feature map, as shown in Figure 4. To extract all local windows, we scan all possible spatial locations of the input feature map. These local windows have the same dimension as the convolutional filters.

The convolution operation is transformed into a matrix multiplication. Therefore, each local window is unrolled into a vector, and then all local windows are grouped in a single matrix. We also unroll each filter to a vector. Note that reshaping the fillers and local windows to vectors need to be done in the same direction to keep the same results of the multiplication operation.

We repeat the previous operations on all filters and input feature maps, then concatenate the vectorized version of all local windows, all filters in the first dimension, and the second dimension, sequentially. Suppose that we have *K* filters; their vectorized version is gathered in a single matrix of size $S_{f}\times K$, where $S_{f}=height\times width\times depth$ of the convolutional filters. Moreover, the input feature maps should be gathered in a single matrix of size $S_{x}\times S_{f}$, where $S_{x}$ represents the number of all possible local windows in all input feature maps. We call this matrix data matrix and refer to it as X. The matrix that contains the filters is denoted as $F_{mat}$.

At this stage, the most important step in the proposed algorithm is to learn the filters. We were inspired by the ELM based auto-encoding approach [12,13] to perform the convolutional filters training. However, many changes have been made to get better results; for example, instead of reconstructing the input, we reconstruct the normalized input. Therefore, first, the data matrix is normalized to have 0 mean, and 1 as standard deviation, referred as $X_{N}$. Second, to handle the convolution bias, we add the intercept term, and learn to reconstruct both the normalized input and the intercept term, so that the target matrix becomes $T=\left[\begin{array}{ccc} X_{N} & | & 1 \end{array}\right]$.

Given the input X and target matrix T, we can apply the ELM-AE algorithm to compute the output weights β. Then, the CONV-layer parameters can be computed as $\left[\begin{array}{ccc} F_{mat}^{T} & | & B^{T} \end{array}\right]=\beta$, where B is the bias vector, which is defined as the transpose of the last column of β. Finally, we just need to reshape the $F_{mat}$ matrix to obtain the filters F. This procedure is summarized in Algorithm 3.

**Algorithm 3** Training procedure of CONV layer using ELM.**Input:** Input feature map. **Output:** CONV parameters: filters F and bias B. Generate normalized training data $X_{N}$. Compute desired target $T=\left[\begin{array}{ccc} X_{N} & | & 1 \end{array}\right]$. Generate randomly the input weights and biases W and b. Compute the hidden matrix $H=G\left(XW+b\right)$. Compute the output weights $\beta =H^{†}T$. Compute filters and bias $\left[\begin{array}{ccc} F_{mat}^{T} & | & B^{T} \end{array}\right]=\beta$. Reshape the filters matrix $F=reshape\left(F_{mat}\right)$. Return CONV parameters F and B.

$X_{N}$ is an $S_{x}\times S_{f}$ matrix that represents the normalized version of data matrix X, where $S_{f}=height\times width\times depth$ of the convolutional filters, and $S_{x}$ represents the number of all possible local windows in all input feature maps of all training examples. As illustrated in Figure 3, X is the data matrix that gathers the vectorized version of all possible local windows generated from the input feature maps of all training examples. T is an $S_{x}\times \left(S_{f}+1\right)$ matrix that represents the target matrix used in the ELM based auto-encoder algorithm to learn the convolutional parameters by reconstructing the normalized input and the intercept term. W is an $S_{f}\times K$ matrix that represents the input weights in the ELM-AE algorithm, where *K* represents the number of filters in the convolutional layer. b is an $S_{x}\times K$ matrix constructed by duplicating the bias vector in the first dimension. In our implementation, W and b are randomly generated according to the normal distribution. G(·) is the activation function of the hidden neurons in the ELM-AE algorithm; in our work, we have used the sigmoid function. H is an $S_{x}\times K$ matrix that represents the hidden matrix, while $H^{†}$ is the Moore–Penrose generalized inverse of the matrix H. β is a $K\times (S_{f}+1)$ matrix that serves as the output weights matrix. The matrix β contains the filters matrix $F_{mat}$, which is an $S_{f}\times K$ matrix that gathers the vectorized version of the convolutional filters, and the bias of the convolutional layer B, which is defined as the transpose of the last column of β. Finally, to obtain the convolutional filters, we just need to reshape the filters matrix to the correct form height×width×depth×K.

The ELM-CNN algorithm is a layer-wise training procedure, which means that we can stop at any desired layer and obtain a model that captures the corresponding features. This is not possible in the case of BP-CNN, where we must train the whole model by back propagating the classification error, and then the desired features can be extracted. In the case of ELM-CNN, if we desire a classification model, we just need to replace the last layer (classification scores) with any classifier, e.g., SVM, ELM, and many others. However, in our algorithm, to compute the final layer output parameters, we minimize the classification error using the same method as computing the output weights of ELM algorithm (based on the Moore–Penrose generalized inverse).

## 3. Experimental Results

The experimental evaluation is divided into three folds. First, we evaluate the features extraction module using the ELM-CNN algorithm. Then, classification ensemble based on extreme learning machine is evaluated and compared with several different algorithms. Finally, the proposed approach that combines the ELM-CNN based features extraction and the ELM based ensemble for classification is evaluated and compared with state-of-the-art models. However, we start this section with a description of the general experimental settings used in our experiments.

### 3.1. Experimental Settings

#### 3.1.1. VAIS Dataset

**Figure 5:**
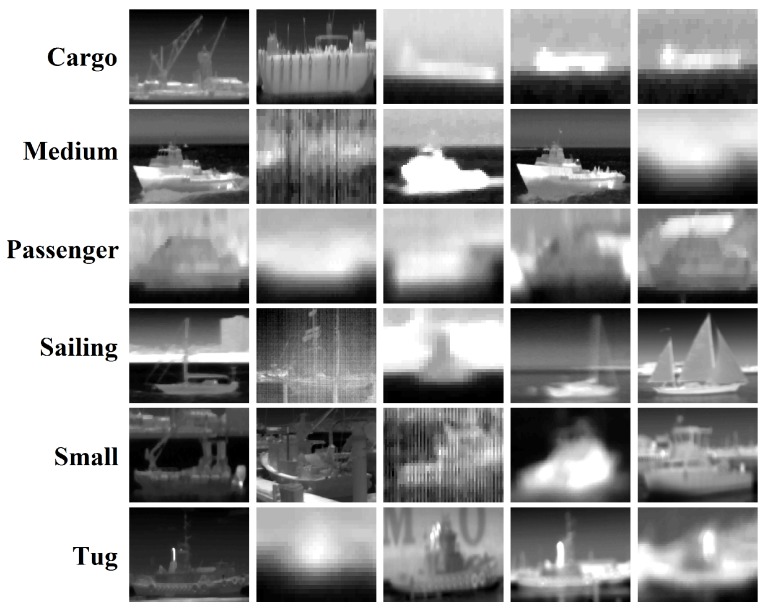
Some training examples of VAIS dataset, each row corresponds to the specified class.

In our experiments, we used the VAIS dataset, which is a publicly available dataset formed by infrared (long-wavelength infrared (LWIR)) images and visible images of ships acquired from piers, suitable for object classification research [1]. Each image was manually annotated to one of six categories (cargo, sailing, passenger, medium, tug, and small). Some training examples are illustrated in Figure 5. In our research, we are interested in object recognition systems that work during both the day and night. Therefore, we only used the infrared images, and this results in a set of 539 training examples and 703 test examples.

#### 3.1.2. Data Pre-Processing

All infrared images from VAIS dataset were re-sized to have the dimension of 79×79 using the Bicubic interpolation method. Then, we subtract the mean image, which is computed as the mean values for each pixel across all training samples. No other pre-processing technique was applied in our experiments.

#### 3.1.3. CNN Architectures

The extreme learning machine algorithm achieves better performance when the number of nodes is sufficiently large. Thus, in our experiments, we adopt two different CNN architectures, as described in Table 1, one small (about $0.43\times 10^{6}$ parameters) and one large network (about $35\times 10^{6}$ parameters). This choice was to: (1) evaluate the performance of ELM-CNN in the case of both large and small networks; and (2) for a fairer comparison between ELM-CNN and BP-CNN, which generally suffers from the problem of overfitting in the case of large networks, particularly when the amount of training data is insufficient, and also a small architecture (less learnable parameters) may reduce the effect of overfitting.

#### 3.1.4. Simulation Environment

We used MATLAB 2016a running on a desktop computer with Intel i7 CPU and 32GB of memory (RAM). For the training of CNN based on back-propagation, we employed the MatConvNet library, which is a MATLAB toolbox of CPU and GPU implementation of Convolutional Neural Networks [16].

### 3.2. Evaluation of Features Extraction Component

In this experiment, we evaluate the performance of features extraction module of the proposed approach. To only measure the effect of features and the influence of learning algorithm used to train the convolutional structure, we provide a comparison based on an independent classifier, which is the linear Support Vector Machine (SVM). First, the CNN features are extracted using one of the two algorithms, ELM-CNN or BP-CNN, and then they are classified using the SVM.

#### 3.2.1. Settings

For the training of the SVM classifier, we have used the LIBLINEAR Library [17], and for parameters selection, five-fold cross-validation method is adopted. In the BP-CNN algorithm, the stochastic gradient descent with momentum is used for parameters updates. The training parameters are set as follows; the momentum is fixed to 0.9, and the batch size is set to 100. In the case of small architecture, the number of training epochs equals 30, the learning rate is fixed to 0.001 for the first fifteen epochs, and then it is divided by 100 for the last fifteen epochs. In the case of the large network, the number of training epochs equals 20, the learning rate is fixed to 0.001 for the first five epochs, and then it is divided by 10 every 5 epochs. In addition, no improvement in the performance is observed when the number of training epochs is increased, and techniques such as dropout are employed to prevent overfitting, but, unfortunately, the performance decreases.

#### 3.2.2. Results

The test accuracy and the training time of both ELM-CNN and BP-CNN algorithms are reported in Table 2 for different types of features captured using the large network. The test accuracy is defined as the accuracy achieved by the SVM classifier on the test set, while the training time is measured as the time required to train the model that can be utilized for features extraction.

To extract any features of interest in the case of the BP-CNN algorithm, we first need to train the classification model. Thus, the training time of BP-CNN is the same for all types of features, which is the time required to train the classification model. In contrast, the proposed ELM-CNN is a layer-wise learning algorithm; therefore, to compute any features of interest, we only need to train the layers that correspond to the desired features. In this context, the training time is defined as the time to train the corresponding layers that will be used to extract the features of interest, so the training time increases for high-level features because more layers must be trained.

The results illustrated in Table 2 show that the ELM-CNN algorithm outperforms the traditional BP-CNN algorithm in term of both generalization performance and training time. In fact, according to our experiments and the used software tools, the ELM-CNN algorithm is at least 150 times faster than the BP-CNN, and, for low-level features, it may be more than 950 times faster.

Extreme learning machines perform properly when the number of hidden nodes is large, however, for back-propagation, it is not the case. A large number of parameters generally leads to overfitting especially when the amount of available data is small like the case of VAIS dataset. To eliminate the hypothesis that may say that the poor performance of BP-CNN is due to the large network not to the small dataset, we propose to repeat the same experiment but with a small network and provide a comparison between the ELM-CNN and BP-CNN features as shown in Table 3.

The results in Table 3 show that, for a small network (about a half million parameters), the ELM-CNN algorithm also exceeds the BP-CNN in term of test accuracy and training speed. In this case, the BP-CNN results are similar to the large network results, except for the speed due to the network size. However, the ELM-CNN algorithm is still faster than BP-CNN (about 55–120 times faster) and, surprisingly, achieves good generalization performance, even for the small network.

These experimental results confirm that the ELM-CNN algorithm outperforms the BP-CNN for both small and large networks with respect to the test accuracy and training speed. The poor generalization performance of the traditional BP-CNN can be interpreted by the overfitting due to the lack of training data in the case of VAIS dataset. Therefore, to make a fair comparison between the ELM-CNN and BP-CNN algorithms, a favorable environment for back-propagation is also demanded. Thus, we propose a comparison on the MNIST dataset, which is a publicly available standard classification benchmark of gray-scale images of handwritten digits $\left\{0,1,\cdots ,9\right\}$. It contains a training set of 60,000 examples and a test set of 10,000 examples. Such amount of training data creates excellent conditions for training the CNN architecture using the BP-CNN algorithm. Moreover, this experiment will be a good opportunity to test the performance of the ELM-CNN approach on a large dataset. We have used the same architecture as the small network, described in Table 1, with stride equals 1 to fit the input images size 28×28. The same training parameters are used except for the learning rate, which is set to 0.001 for all the 20 training epochs. The results of the experiments performed on the MNIST dataset are presented in Table 4.

The results presented in Table 2 and Table 3 show that the ELM-CNN algorithm performs better than BP-CNN in the case of VAIS dataset. This conclusion might still be true for small datasets, but it could not be generalized to large datasets, where BP-CNN still performs better, as provided in the MNIST example in Table 4. However, the ELM-CNN approach achieves competitive results in term of generalization performs especially for low-level features, as for the training speed, the ELM-CNN algorithm is 5–16 times faster than the BP-CNN. Therefore, the proposed approach, ELM-CNN, is still a good alternative of BP-CNN, particularly when fast training is required in large datasets, while, for small datasets, the ELM-CNN algorithm may be considered as the best alternative to train any convolutional architecture.

#### 3.2.3. Features Fusion Results

The purpose of this experiment was to test the influence of features fusion on the generalization performance on VAIS dataset. We have used the small network architecture, and the fused features vector is computed as the concatenation of two features types. The test accuracy of the SVM classifier is reported in Table 5.

There is no clear conclusion that the fusion of features improves the generalization performance. In fact, sometimes the result is worse, which may be interpreted by the features poor quality due to the lack of training data in the case of VAIS dataset. It seems that those features contain some undesired information such as noise. Reducing the dimension of features using some dimensionality reduction techniques may improve the performance, but this is not the purpose of this paper. For this reason, in our proposed approach, we only used one features type and fed it to our proposed ELM based ensemble for classification, which will be evaluated in the next section.

### 3.3. Evaluation of the Classification Component

The second component of our approach is the ELM based ensemble for classification. As explained in Section 2.3, the ELM based ensemble is capable of performing any classification problem. Therefore, in this section, we provide an evaluation of the proposed ensemble on several standard classification benchmarks, specified in Table 6. All datasets are taken from LIBSVM Data and UCI Machine Learning Repository except the VAIS dataset. A comparison with several classification algorithms is carried out in term of generalization performance and training speed.

For the experimental settings, we used the linear SVM, k-Nearest Neighbors KNN (#neighbors = 10, Euclidean distance metric), Decision Trees DT (maximum #splits = 20, Gini’s diversity index criterion), an ensemble of boosted DT (AdaBoost algorithm, #learners = 30, learning rate = 0.1), and a bootstrap-aggregated ensemble bagged DT (bag method, #learners = 30).

Table 7 shows the test accuracy and the training time of different classifiers on several real classification problems. The results confirm that the proposed ELM based ensemble is very fast in training and achieves high generalization performance compared to some traditional classifiers, such as SVM and KNN, and ensembles, such as boosted DT, and Bagged DT. For example, the ELM based ensemble improves the generalization performance by more than 16% and 23%, compared to Bagged DT in the case of Duke dataset, and SVM in the case of Hill dataset, respectively. As for the training speed, the proposed ensemble is seven and four times faster than Bagged DT on Duke dataset, and SVM on Hill dataset, respectively.

### 3.4. Evaluation of the Proposed Approach

After evaluating each component separately, in this section, we evaluate the whole proposed approach that combines the features extraction using the ELM-CNN algorithm and the ELM based ensemble for classification. We tested different types of features and used both the small and large networks and compared the results with state-of-the-art methods on the VAIS dataset, in term of generalization performance. We also reported the hyperparameters of the used ELM based ensemble; *M* the number of individual ELM models, and *L* the number of hidden nodes, which are determined by five-fold cross-validation method.

In [1], three approaches were reported; first, the Gnostic Field algorithm trained on features computed as a combination of SIFT (Scale-invariant feature transform) descriptors and a 5-d vector that represents the spatial location information, and its dimension is reduced using the whitened PCA (Principal Component Analysis). Second, the CNN architecture, where a large pre-trained CNN (15 layers) is used to extract features then classified using the logistic regression. Third, a combination of the two methods is used by averaging of the output of each classifier.

The results of Table 8 show clearly that almost all models of our proposed approach outperform the three approaches developed in [1]. The best result is achieved when using CONV_3 features computed using the large network. Moreover, the performance of the small network is also better than the Gnostic Field, CNN, and their combination. Therefore, the main conclusion of this experiment is that the proposed approach outperforms the state-of-the-art models of VAIS dataset.

## 4. Discussion

The proposed approach is built on two components: features extraction using the ELM-CNN algorithm and classification using the ELM based ensemble. Each component has been evaluated separately, and the overall evaluation is also carried out by comparing with different methods. Accordingly, in this section, we discuss the presented results in the context of existing works and try to provide some interpretations that may highlight new perspectives for future work.

### 4.1. The ELM-CNN Approach

We first start with the ELM-CNN algorithm, which is a new framework based on extreme learning machines able to train any convolution neural network. The proposed algorithm employs the idea of extreme learning machine based auto-encoding [12,13]. However, our proposed method ELM-CNN improves the performance and can handle the bias of convolutional filters by reconstructing the normalized input and the intercept term. Moreover, the results shown in this paper confirm that the ELM-CNN is capable of training both small and large networks. The comparison with the traditional back-propagation training of convolutional neural networks shows that the ELM-CNN outperforms the BP-CNN in term of generalization performance and training speed, especially when the available amount of training data is small, which is the case of infrared-based object recognition research such as VAIS dataset. Note that, to collect the data of the VAIS dataset, more than nine days have been spent plus the manual annotations to produce just a few hundred infrared images [1]. This is still a small dataset compared to millions of visible images in the case of ImageNet [5], which triggered the success of convolutional neural networks. Hence, according to the results presented in this paper and the difficulty to build a large dataset, the ELM-CNN algorithm may be the best alternative to train CNN, particularly in the case of infrared images, and small datasets, especially when the BP-CNN suffers from the problem of overfitting due to the lack of training data. The MNIST example, presented in Section 3.2.2, shows that, when enough training examples are available, the ELM-CNN algorithm also achieves competitive results in term of generalization performance compared to the BP-CNN. As for the training speed, the ELM-CNN algorithm still much faster.

The advantages of ELM-CNN training method are not just limited to the generalization performance and speed, but also the ELM-CNN is an unsupervised algorithm, therefore no need for labels to train the CNN architecture, unlike the BP-CNN that requires the classification model to extract any desired CNN features. In addition, the ELM-CNN is a layer-wise training algorithm, which means that, to extract any features of interest, only the corresponding layers require to be trained, which explains the training speed of the ELM-CNN. In fact, for low-level features, the ELM-CNN is up to 900 faster than the BP-CNN algorithm, as shown in Table 2. The strengths of the ELM-CNN algorithm, especially the training speed, may elicit some applications like pre-training and hyperparameters search. Indeed, the ELM-CNN can be used to pre-train a convolutional neural network, then fine-tuned using the back-propagation algorithm. This multistage training procedure may lead to better generalization performance with a small training time compared to BP-CNN alone. In addition, the ELM-CNN can be used for search of the best network structure parameters such as the number of layers, the number of kernels, and the size of filters. Then, after finding the best parameters, the BP-CNN algorithm can be used for training.

### 4.2. The ELM Based Ensemble

**Figure 6:**
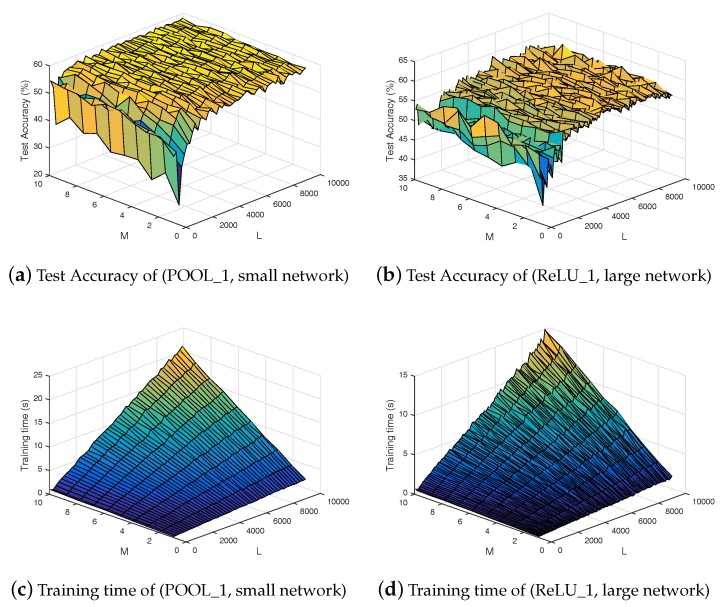
Analysis of the ELM based ensemble with respect to # individual models *M* and # hidden nodes *L* on VAIS dataset. Test Accuracy in the case of: (**a**) POOL_1 features using the small network; and (**b**) ReLU_1 features using the large network. The training time in the case of: (**c**) POOL_1 features using the small network; and (**d**) ReLU_1 features using the large network.

After extracting useful information using the ELM-CNN features extraction component, the second step of the proposed approach is the classification using the ELM based ensemble. The proposed ELM based ensemble is a fully learnable framework that learns automatically how to combine different classifiers by minimizing the classification error and the norm weights, which leads to better generalization performance. The comparison with several methods; standard classifiers like SVM and KNN, ensembles like Bagged DT and Boosted DT shows that the developed model achieves better results with respect to the test accuracy and training time. For example, the ELM based ensemble is 8% better and five times faster than the SVM classifier in the case of Sonar dataset. These results can be interpreted by the fact that an ensemble with plurality consensus always performs better than a single model [18], and also minimizing the norm of network weights (output weights of each classifier and the combination weights) leads to better generalization performance according to Bartlett’s theory [14]. As described in Section 2.3, the proposed ensemble can perform any classification task, and also it can be adapted to perform the regression task and the online sequential learning in a similar way to the ensemble of online sequential ELM [19]. The ELM based ensemble has only two hyperparameters, which can be determined using cross-validation method; *M* the number of models used in the ensemble, and *L* the number of hidden nodes for each ELM. The influence of these parameters with respect to the generalization performance and training time is illustrated in Figure 6. Note that all the *M* individual ELM models share the same number of hidden nodes *L* and the same activation function G(·).

According to the results presented in Figure 6, a large set of configurations (*M* and *L*) can be used to obtain similar generalization performance. The ELM based ensemble is a fast learning algorithm, so the user may run a grid search to fit the best hyperparameters. However, to balance between the generalization performance and the training speed, we recommend using a medium number of models and an average number of nodes depending on the size of the classification problem.

### 4.3. The Proposed Approach

The proposed approach is fully based on extreme learning machines; indeed, it combines the ELM-CNN based features extraction and the ELM based ensemble for classification. Therefore, the proposed approach inherits from the ELM algorithm the speed and the generalization performance, which makes it outperforms the state-of-the-art methods of VAIS dataset [1], and the comparison results illustrated in Section 3.4 confirm these conclusions. After analyzing the different methods presented in [1], we found that, first, the Gnostic Field algorithm is trained on intermediate features that combine the SIFT descriptors and a 5-d vector that represents the spatial location information. This is not provided in the public VAIS dataset, which favors the Gnostic Field algorithm and leads to an unfair comparison. Although this unavailable information is used in Gnostic Field, our approach outperforms it in term of generalization performance, as shown in Table 8. Second, VAIS dataset [1] used the VGG net [20] for features extraction, which is a large network trained on ImageNet dataset [5]. We believe that there are two problems with this approach: (1) The VGG net is trained on visible images which differ from LWIR images that are sensitive to thermal emissions, not light, which is why the VGG net achieves the lowest performance. (2) The VGG net requires an input of dimension 224×224; therefore, VAIS dataset [1] has up-sized all images to conform the input size of VGG net. This means that, for example, if we have an image with an initial size of 56×56, one real pixel become a region of 4×4. Knowing that the VGG net uses a stack of 3×3 convolutional filters, the receptive field of VGG net does not see any real information in the input space (sees $\frac{3}{4}$ of the pixel). This also may contribute to the interpretation of the lowest performance achieved by VGG net in [1].

Third, VAIS dataset [1] proposed a combination of Gnostic Field and VGG net using a weighted average, the value of 0.8 is assigned to the VGG output and weight of 0.2 to the output of Gnostic Field. The principal problem is that there is no explanation and no method to determine these weights are supplied in [1]. However, many different types of features in our proposed approach outperform all the models presented in [1].

To the best of our knowledge, VAIS dataset [1] is the largest dataset of maritime ships in infrared images. However, it still relatively small, which may explain why the high performance could not be achieved. To provide more insights about the VAIS dataset, we analyzed the misclassified samples using our best approach, and investigated different sources of the error, which may provide more reliable interpretation of the performance results.

**Figure 7:**
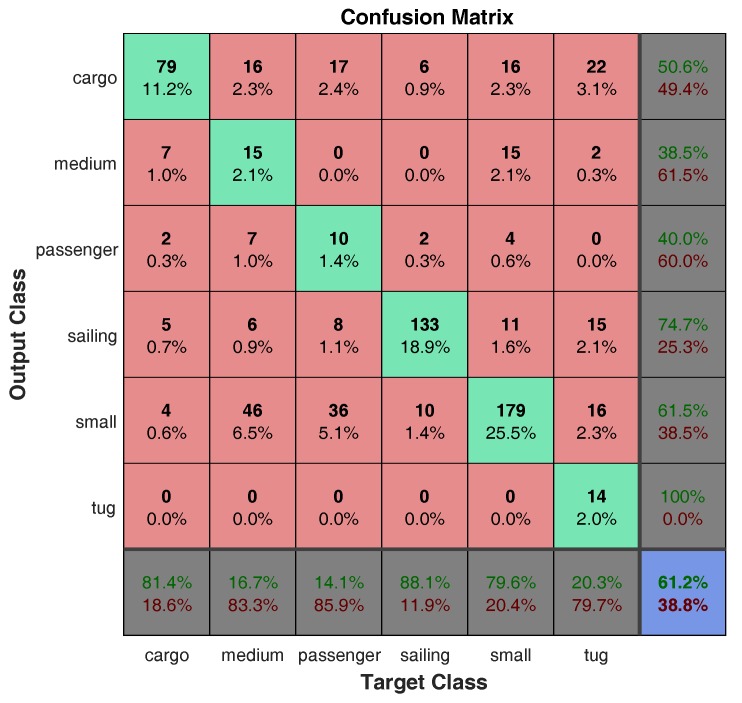
The confusion matrix of our best approach (CONV_3 features, large network).

Figure 7 shows the confusion matrix of the proposed approach that relies on the CONV_3 features computed using the large network. The results show that the most accurate classes are *sailing*, *cargo*, and *small* with accuracy equal to 88.1%, 81.4%, and 79.6%, respectively, while the other classes (*medium*, *passenger*, and *tug*) are the most confused categories with accuracy less than 20%. The examples labeled as *medium* or *passenger* are often misclassified as *small*, and the samples that belong to *tug* category are frequently confused with *cargo*, *small*, or *sailing* classes.

**Figure 8:**
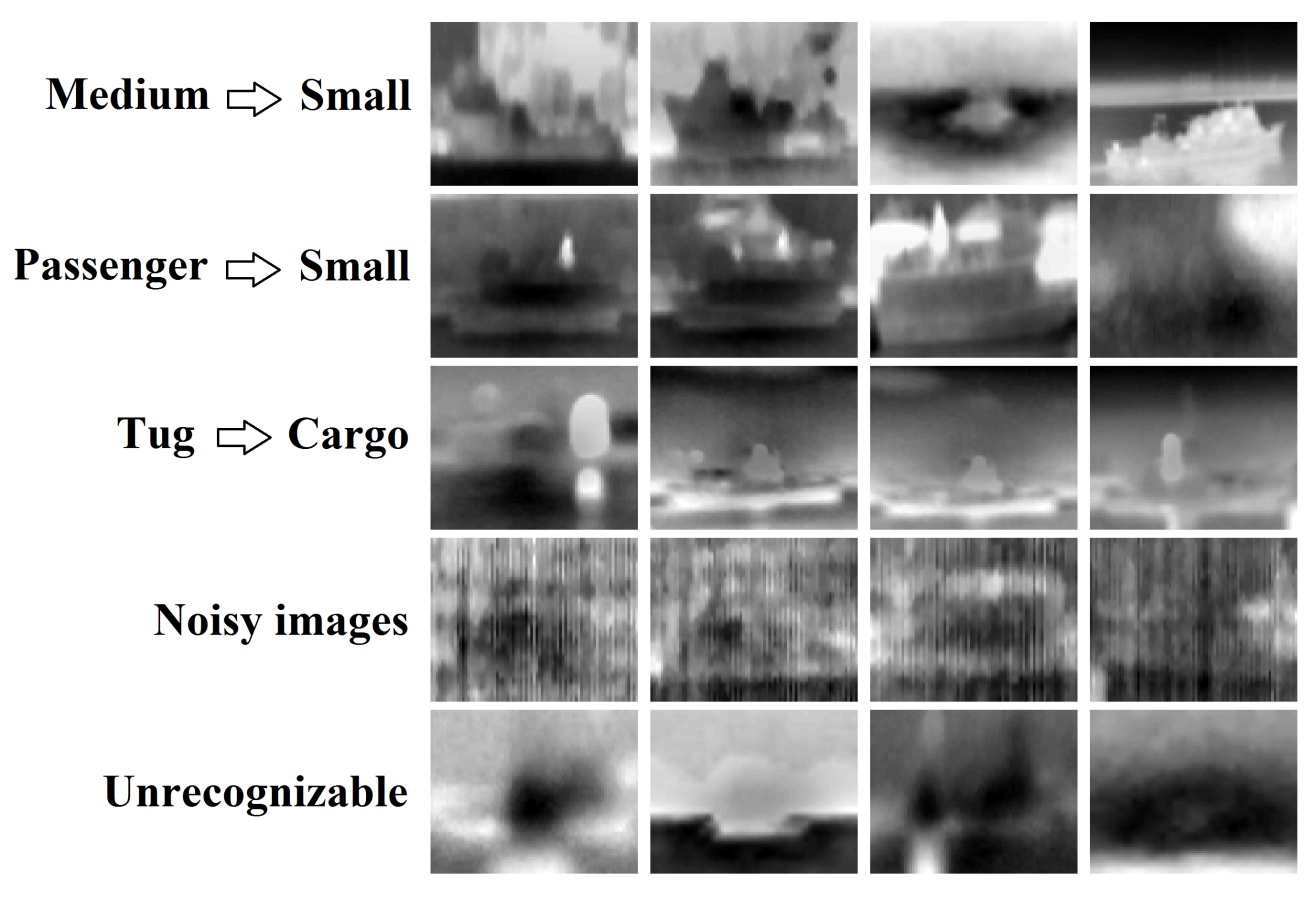
Some misclassified VAIS test samples using our approach (CONV_3 features, large network). The first row, for example, corresponds to some test samples labeled as *Medium* and misclassified by our algorithm as *Small*.

**Figure 9:**
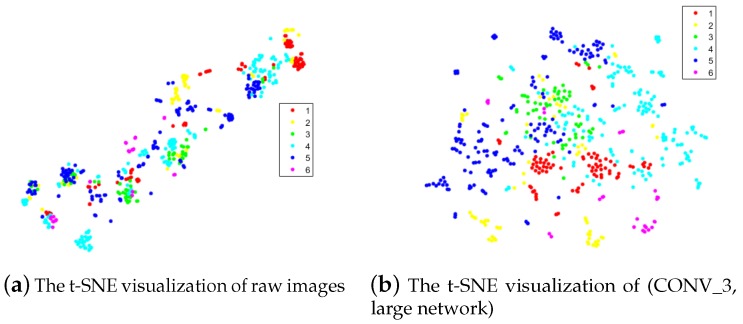
The t-SNE visualization of VAIS test set examples: (**a**) raw images; and (**b**) CONV_3 features using the large network.

To understand more the confusion between the different classes, we visualized all misclassified samples and came up with some explanations, as shown in Figure 8. The first row shows some examples labeled as *medium* and misclassified by our algorithm as *small*. The second row shows some *passenger* samples confused with *small*, while the third row shows some samples of the *tug* category misclassified as *cargo*. According to this visualization, we can see obviously that it is difficult to classify these examples, even for a human, correctly. In addition, some examples are completely unrecognizable as shown in the two last rows of Figure 8, due to the noise or low resolution of images (objects are far from the camera). Therefore, we believe that excluding these unrecognizable images may be a reasonable choice because demanding to an algorithm to correctly classify unrecognizable examples is not meaningful. However, these visualizations also confirm that the VAIS dataset is a very challenging dataset, and the improvement of performance depends on more distinctive features. For a qualitative understanding of how the features are distinctive in our approach, a 2-D visualization is illustrated in Figure 9 using the t-SNE (t-distributed Stochastic Neighbor Embedding) [21], which is a popular tool for visualizing high dimensional data.

Figure 9 shows the t-SNE visualization of raw images and (CONV_3, large network) features on the VAIS test set. Each point is a 2-D embedding of the features codes or the raw images of a particular example. For the raw images, all points that do not belong to the same class are grouped. However, for the CONV_3 features, the points that belong to the same class are near each other but not sufficiently separated, and many overlapping regions exist between different classes. It is clear that CONV_3 features are more distinctive than raw images, and, to learn more distinctive features, more training examples are required. Moreover, improving the performance might rely on building larger datasets; meanwhile, our approach may be still the best alternative, especially for small datasets, although competitive results were achieved in large datasets such as the MNIST dataset.

## 5. Conclusions

In this paper, an approach fully based on extreme learning machine for maritime ships recognition in LWIR images is proposed. In fact, the proposed approach combines the ELM based learning algorithm of convolutional neural networks for useful features extraction and an ELM based ensemble for classification. Each algorithm is evaluated separately and compared with existing works; then, the overall approach is also evaluated and compared with state-of-the-art methods on the VAIS dataset. The experimental outcomes confirm the superiority of our proposed approach in terms of generalization performance and training speed. Due to the lack of training examples in the case of infrared images based object recognition datasets, the proposed ELM-CNN algorithm is the best alternative for features extraction, and also the proposed ELM based ensemble improves further the classification performance.

The extensive analysis of the proposed approach provides some practical guidelines for the users concerning the hyperparameters selection. Moreover, it may highlight some forward perspectives for future research. First, the ELM-CNN algorithm may be applied for pre-training and network structure search. Second, the ELM based ensemble can be adapted for regression and online sequential learning. Third, the overall approach may be generalized to any object recognition problem, especially when the amount of training data is small, where it might be the best alternative.

## Figures and Tables

**Table 1:** Description of the adopted CNN architectures.

Layers	Parameters (Small CNN)	Parameters (Large CNN)
CONV_1	Filters: 5×5×1×20	Filters: 5×5×1×200
	Stride: 2	Stride: 2
POOL_1	Method: max	Method: max
	Size: 2×2	Size: 2×2
	Stride: 2	Stride: 2
CONV_2	Filters: 5×5×20×50	Filters: 5×5×200×500
	Stride: 2	Stride: 2
POOL_2	Method: max	Method: max
	Size: 2×2	Size: 2×2
	Stride: 2	Stride: 2
CONV_3	Filters: 4×4×50×500	Filters: 4×4×500×4000
	Stride: 1	Stride: 1
ReLU_1	Rectified Linear Unit	Rectified Linear Unit
CONV_4	Filters: 1×1×500×6	Filters: 1×1×4000×6
(FC)	Stride: 1	Stride: 1

**Table 2:** Features performance comparison between ELM-CNN and BP-CNN on the VAIS dataset (Large Network).

	Test Accuracy (%)		Training Time (s)	
**Features**	**BP-CNN**	**ELM-CNN**	**BP-CNN**	**ELM-CNN**
POOL_1	53.34	**57.04**	4608.7	**4.8109**
CONV_2	53.49	**54.91**	4608.7	**16.2552**
POOL_2	**54.62**	54.20	4608.7	**17.3252**
CONV_3	55.19	**57.18**	4608.7	**28.3843**
ReLU_1	50.92	**57.33**	4608.7	**30.8163**

**Table 3:** Features performance comparison between ELM-CNN and BP-CNN on the VAIS dataset (Small Network).

	Test Accuracy (%)		Training Time (s)	
**Features**	**BP-CNN**	**ELM-CNN**	**BP-CNN**	**ELM-CNN**
POOL_1	52.06	**57.33**	151.09	**1.2149**
CONV_2	**54.77**	52.77	151.09	**2.1981**
POOL_2	52.20	**58.75**	151.09	**2.2708**
CONV_3	51.78	**55.48**	151.09	**2.6187**
ReLU_1	51.49	**55.48**	151.09	**2.7779**

**Table 4:** Features performance comparison between ELM-CNN and BP-CNN on the MNIST dataset.

	Test Accuracy (%)		Training Time (s)	
**Features**	**BP-CNN**	**ELM-CNN**	**BP-CNN**	**ELM-CNN**
POOL_1	98.27	**98.36**	767.45	**46.90**
CONV_2	**98.01**	97.53	767.45	**118.88**
POOL_2	**98.97**	98.54	767.45	**132.49**
CONV_3	**98.84**	97.53	767.45	**145.33**
ReLU_1	**99.16**	98.09	767.45	**157.08**

**Table 5:** Generalization performance of ELM-CNN features fusion on VAIS dataset.

Features	POOL_1	CONV_2	POOL_2	CONV_3	ReLU_1
**POOL_1**	55.33	51.92	54.48	51.92	51.92
**CONV_2**	57.18	52.77	54.05	52.92	52.63
**POOL_2**	54.48	54.05	58.75	53.63	53.91
**CONV_3**	51.92	52.92	53.63	54.62	55.19
**ReLU_1**	51.92	52.63	53.49	55.33	55.48

**Table 6:** Specifications of classification benchmark datasets.

Datasets	# Atrrib	# Classes	# Train	# Test
Balance	4	3	400	225
DNA	180	3	1400	1186
Duke	7129	2	29	15
Hill	100	2	606	606
Sonar	60	2	150	58
Vais	6241	6	539	703
Waveform	21	3	3000	2000

**Table 7:** Performance comparison of ELM based ensemble with SVM, KNN, DT, Boosted DT, and Bagged DT.

Datasets	SVM		KNN		DT		Boosted DT		Bagged DT		ELM Based Ensemble	
	Accuracy	Time	Accuracy	Time	Accuracy	Time	Accuracy	Time	Accuracy	Time	Accuracy	Time
Balance	$\underline{91.62}$	0.5255	88.88	0.3015	78.86	0.2818	84.1	1.5625	85.5	1.5742	$\underline{91.33}$	0.0023
DNA	$\underline{94.22}$	0.8162	78.04	0.4583	90.78	0.3303	91.86	1.6845	92.7	1.7161	$\underline{94.03}$	0.1287
Duke	78.58	6.3785	78.58	6.6090	77.14	6.5628	50.00	6.6966	78.58	7.8948	95.33	1.0523
Hill	75.04	2.3415	52.08	0.2477	52.10	0.4326	54.24	3.0291	56.94	1.4499	98.59	0.5775
Sonar	76.56	0.2637	75.50	0.2930	73.10	0.3630	53.40	0.4671	83.80	1.0354	85.17	0.0450
Vais	50.07	34.760	47.37	23.722	38.55	33.068	43.95	220.49	43.67	30.603	52.92	2.5819
Waveform	$\underline{86.64}$	1.0923	82.9	0.3081	75.22	0.3091	81.4	2.1960	84.16	1.9354	$\underline{86.55}$	0.0499

**Table 8:** Comparison with state-of-the-art approaches on the VAIS dataset (IR images only).

Approaches	Test Accuracy (%)	M	L
Gnostic Field [1]	57.21	-	-
CNN [1]	55.29	-	-
Gnostic Field + CNN [1]	57.72	-	-
POOL_2 (Large Net)	56.76	6	100
CONV_3 (Large Net)	**61.17**	1	9000
ReLU_1 (Large Net)	60.73	8	9000
POOL_1 (Small Net)	59.60	8	2900
POOL_2 (Small Net)	58.03	4	100
ReLU_1 (Small Net)	57.89	3	10900

M: # individual ELMs; L: # hidden nodes.
